# Hepatic Endometriosis Misdiagnosed as Hepatic Epithelioid Hemangioendothelioma

**DOI:** 10.1155/crhe/3676537

**Published:** 2025-08-15

**Authors:** Aditya Kler, Nirjhar Dutta, Jennifer Haglund

**Affiliations:** ^1^Division of Hospital Medicine, Department of Medicine, University of Maryland, Baltimore, Maryland, USA; ^2^Division of Gastroenterology, Hepatology, and Nutrition, Department of Medicine, University of Minnesota, Minneapolis, Minnesota, USA; ^3^Division of Gastroenterology, Hepatology, and Nutrition, Department of Medicine, Veterans Administration Medical Center, Minneapolis, Minnesota, USA

## Abstract

We describe the case of a young 42-year-old female who suffered from cyclical right upper quadrant abdominal pain that led to an emergency department visit where an intrahepatic mass was found on imaging. Initial CT-guided biopsy led to the diagnosis of hepatic epithelioid hemangioendothelioma, and the patient was referred to a liver transplant center. She underwent hepatic segmentectomy with right nephrectomy and surgical pathology came to the correct diagnosis of hepatic endometriosis.

## 1. Introduction

Endometriosis affects 10% of women in their reproductive age [1, 2]. Hepatic endometriosis (HE) has been described only a handful of times in the published literature [3]. Diagnosis of HE is difficult as patients rarely present with classical cyclical pain related to menses.

## 2. Case Presentation

A 42-year-old female veteran presented to the emergency department (ED) for acute right upper quadrant abdominal pain. The patient had been having intermittent right upper quadrant pain for a few months prior to this ED presentation, which she thought was related to the consumption of gluten-containing foods. Pain would last, she did not identify any other trigger for the pain, and the pain was not always related to the menstrual cycle. Her medical history included obesity, hyperlipidemia, and Graves' disease status post-radioactive iodine therapy, on thyroid hormone replacement therapy, seasonal allergies, and non-Celiac gluten sensitivity. Surgical history was notable for cholecystectomy for biliary dyskinesia. She had been pregnant four times and had given birth to four healthy children, the last one 3 years prior to this ED visit. She had an ultrasound of her liver done in January 2022 due to an incidentally noted liver cyst noted on the MRI of lumbar spine for sciatic pain, and that ultrasound showed normal liver size and echotexture with a 1.1 × 1.3 cm anechoic lesion in the right lobe of the liver without vascularity representing a cyst. There was no mass/cyst noted in the right kidney. Liver lab tests at that time were the following: AST 28 U/L, ALT 21 U/L, alkaline phosphatase 74 U/L, total bilirubin 0.5 mg/dL, white blood cell count 5.6 K/cmm, hemoglobin 13.2 g/dL, platelet count 224 K/cmm, and albumin 4.4 g/dL.

**Figure 1:**
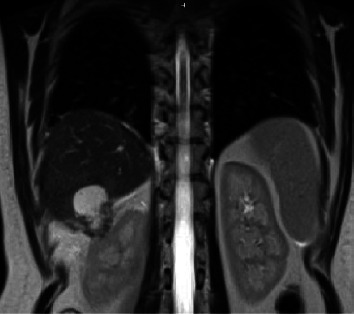
MRI showing RUQ complex cystic mass in the liver extending into the R renal fossa.

In the ED, CT scan of abdomen and pelvis with IV contrast showed a complex cystic mass in the right upper quadrant extending into the renal fossa. The patient subsequently underwent an outpatient hepatology consultation and outpatient MRI abdomen with gadolinium (Figure 1). The MRI showed a complex septate enhancing cystic lesion in segment VI with extrahepatic extension into the pararenal soft tissues measuring 3.5 × 3.4 × 4.7 cm, causing mass effect on the upper pole of the right kidney. Differential diagnosis included an infected cyst/abscess, such as hydatid cyst, and mucinous cystic neoplasm. A simple left hepatic lobe cyst measuring 3.6 cm was also noted. Liver was otherwise noted to be noncirrhotic and without fatty changes, without biliary dilation. The GI tract, spleen, pancreas, adrenals, and bilateral kidneys were otherwise noted to be unremarkable.

Liver tests showed the following: AST 22 U/L, ALT 26 U/L, alkaline phosphatase 75 U/L, total bilirubin 0.3 mg/dL, white blood cell count 5.8 K/mL, hemoglobin 12.7 g/dL, platelet count 222 K/mL, albumin 4.3 g/dL, and INR 1.0. A CT-guided core biopsy was performed. Infectious workup—including bacterial, fungal, and parasitic infections—was negative. Serological tests for hepatitis A, B, and C were negative, and serum tumor marker testing revealed a slightly elevated CA 19-9 level (76 U/mL, reference < 25) along with normal AFP and CEA. Histopathological examination, which was done locally and at another tertiary care center for a second opinion, was reported as “fragments of fibroconnective and adipose tissue with nonspecific inflammatory changes and fibrosis, negative for neoplasm.” Immunohistochemical analysis showed CD68 positivity, indicating histiocytic inflammation, and CD34 positivity, highlighting vascular components; cytokeratin AE1/AE3 staining was negative, which excluded metastatic carcinoma. Erythroblastosis transformation-specific regulated gene 1(ERG) staining was weak to moderately positive, and the second opinion on the histology raised the question of “this cytology seems most suggestive of a fibrous capsule or reaction to abscess or other lesion, however ERG staining raises the differential of hemangioendothelioma or similar lesion.”

Given the diagnostic uncertainty and given hepatic epithelioid hemangioendothelioma (HEHE) was in the differential diagnosis, which has malignant potential, the patient was referred to a liver transplant center for consideration of primary resection versus orthotopic liver transplantation based on the extent of the suspected, which qualifies for a standardized model for end-stage liver disease (MELD) exception [4]. The case was discussed in a multidisciplinary tumor conference with hepatology, radiology, medical oncology, and surgical oncology, and the differential was narrowed down to fibrous reaction to prior abscess, soft tissue mass with internal hemorrhage, and less likely classical HEHE, and a plan was made to proceed with complete surgical resection.

**Figure 2:**
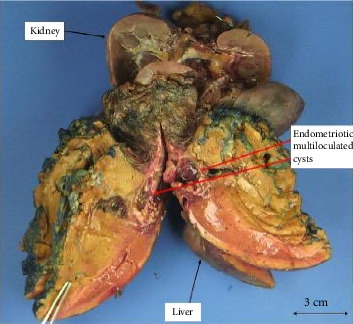
Gross specimen of surgical explant with portions of liver, diaphragm, and right kidney showing endometriotic multiloculated cysts.

**Figure 3:**
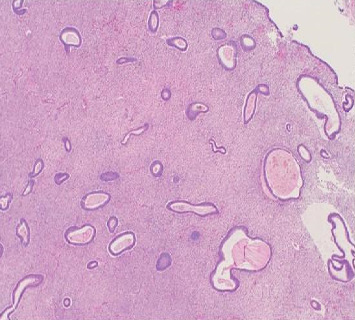
Hematoxylin and eosin staining in high-power magnification to illustrate endometrial tissue forming stroma with menstrual sloughing in the interior.

The patient subsequently underwent hepatic segmentectomy and right nephrectomy. Gross examination revealed multiloculated cysts consistent with endometriosis (Figure 2). Final surgical pathology confirmed HE, demonstrating endometrial glands and stroma without dysplasia or malignancy (Figure 3).

The patient recovered quickly and is now asymptomatic.

## 3. Discussion

This case highlights the presentation of HE. HE is a rare form of endometriosis characterized by ectopic endometrial tissue in the liver. It typically presents with right upper quadrant pain. In some cases, the pain may be cyclical with menstruation. Diagnosis is challenging due to the rarity of the disease and nonspecific clinical and radiological findings, thus typically requiring histopathological confirmation. MRI and CT may reveal cystic liver masses, but definitive diagnosis usually necessitates surgical resection. While preoperative biopsy may assist in diagnosis, it carries risks.

Various theories have been proposed regarding the pathophysiology of endometriosis with the leading ones being retrograde menstrual blood flow seeding endometrial tissue into the peritoneal cavity versus germ cell mutations or vascular and lymphatic spread [5]. First-line therapy for endometriosis is hormonal contraceptives and pain control. Surgical intervention, such as laparoscopic excision, is reserved for the failure of medical management or diagnostic uncertainty [6]. One systematic review of HE patients identified 32 patients (mean age 39, 62% nulliparous and 75% of reproductive age); only about a third of the patients had a prior diagnosis of pelvic endometriosis, and the following were their surgical resections: cyst resection and minor and major liver resections were performed in 14/31, 9/31, and 8/31 patients, respectively [7]. Of note, there is a documented risk of malignant transformation within endometriotic tissue, with cases of sarcoma and adenocarcinoma arising from HE [8, 9].

HEHE is a rare vascular tumor that originates from endothelial cells and is felt to have malignant potential, which is why this diagnosis grants patients a pathway to liver transplantation with an exception to the MELD score. Its clinical presentation varies considerably, ranging from incidental detection on imaging to nonspecific symptoms like abdominal pain and weight loss. CT scan may show subcapsular localization, capsular retraction, and calcifications. Characteristic radiologic signs such as the “lollipop” and “target” signs can be observed on CT or MRI [10]. HEHE generally has an excellent prognosis and is considered curable. Long-term outcome studies suggest that surgical resection is the preferred treatment for localized or single lobe disease, achieving cure rates of 70%–80% [11]. Liver transplantation remains the preferred option for bilobar disease with the 5-year survival rate of around 77%, generally comparable to the 5-year overall survival rate for liver transplant [12, 13]. According to one study from the National Cancer Database in the United States, most HEHE patients (65.2%) were managed nonoperatively, while 22.1% underwent hepatic resection and only 2.1% received liver transplants [14].

Our case highlights the diagnostic challenges associated with both HE and HEHEs. To help clarify the differences between these two disease entities, key features are shown in Table 1. In conclusion, HE and HEHEs are two rare diagnoses that should be on the differential diagnosis of atypical liver lesions in a patient assigned female at birth.

## Figures and Tables

**Table 1:** Key features highlighting differences between hepatic endometriosis and hepatic epithelioid hemangioendothelioma.

Feature	Hepatic endometriosis	Hepatic epithelioid hemangioendothelioma
Etiology	Ectopic endometrial tissue [6]	Vascular endothelial cell origin [10]
Incidence	Extremely rare, with only about 21 cases reported [15]	Rare, with an incidence of 1-2 cases per million [10]
Age at diagnosis	Typically, women of reproductive age but can occur after menopause [6]	40–50s [10]
Clinical presentation	Abdominal pain, often cyclical and related to the menstrual cycle; infertility [6]	Nonspecific symptoms (e.g., right upper quadrant pain and weight loss) but more commonly incidental finding [10]
Imaging characteristics	May present as cystic liver lesions [16]	Hypoechoic, heterogeneous on US; subcapsular location, capsular retraction, calcifications on CT/MRI [10]
Histopathology	Endometrial glands and stroma (CD10, CK7), estrogen/progesterone receptors [17]	Epithelioid and histiocytoid cells, positive for endothelial markers (CD31, CD34, factor VIII-related antigen) [18]
Genetic markers	Nonspecific [6]	WWTR1–CAMTA1 fusion, YAP1–TFE3 fusion [19]
Treatment	Hormonal therapy, surgical resection [6]	Surgical resection, liver transplantation, ablative therapies, systemic therapy in select cases [10]
Prognosis	Generally good and centered on symptom management [6]	Variable; median survival 15 years, poor prognosis with lung/multiorgan involvement, ascites, age > 55, male gender [10]

## Data Availability

The data supporting the findings of this study are available from the corresponding author upon reasonable request.
